# The importance of the laboratory to the pharmaceutical business

**DOI:** 10.1155/S1463924600000067

**Published:** 2000

**Authors:** Juanita M. Hawkins

**Affiliations:** Janssen Pharmaceutica Titusville NJ USA

## Abstract

Understanding the contributions that the laboratory can make in product/process development, process improvement, market surveil lance and general business is key to the pharmaceutical business today. Poor laboratory practice yields compliance issues, increased cost, increased cycle time and delayed product introductions. This paper covers key areas of customer satisfaction, the role of quality control and quality assurance laboratories, measures of account ability and progress, and an example of how laboratory robotics can help meet important goals.


Journal of Automated Methods & Management in Chemistry, Vol. 22, No. 2 (March–April 2000) pp. 47–52

### The importance of the laboratory to the pharmaceutical business

**Juanita M. Hawkins**

*Janssen Pharmaceutica, Titusville, NJ, USA*

*Understanding the contributions that the laboratory can make in product/process development, process improvement, market surveillance and general business is key to the pharmaceutical business today. Poor laboratory practice yields compliance issues, increased cost, increased cycle time and delayed product introductions. This paper covers key areas of customer satisfaction, the role of quality control and quality assurance laboratories, measures of accountability and progress, and an example of how laboratory robotics can help meet important goals.*

### Introduction

The objective of this paper is to prompt thought and discussion on the work of analytical laboratories, and their contribution and importance to the business. Firstly, a brief background of Janssen Pharmaceutica is given to establish a point of comparison to other companies and businesses.

- Janssen Pharmaceutica is the pharmaceutical sector of Johnson & Johnson.
- It has sales in excess of £ 1.5 billion.
- Its headquarters are in Belgium.
- It produces primarily for USA.
- It competes internally and externally.

Janssen Pharmaceutica is a corporate entity which develops policies and dictates and monitors financial and business expectations; the corporate staff provide financial, business and ethics goals. The focus of the company is stated in its Credo, a document established over 75 years ago by its founder. The Credo clearly documents the order of priority: customer; employee; community; and stockholder.

Janssen is unique within J&J in that the franchise headquarters are in Belgium, the production facility is in Gurabo, Puerto Rico, and US administrative and laboratory areas are in New Jersey. This is critical to business success as it mandates recognition and understanding of cultural and regulatory differences. Translated to the laboratory aspects of the business, Janssen's methods and laboratory processes must meet the requirements of key global regulatory authorities, yet play out within the cultural guidelines of the countries supplied.

As one of the J&J family of companies, Janssen is expected to compete internally and externally for best practice and benchmark position.

### Discussion

Janssen's Credo:

- We believe our first responsibility is to the doctors, nurses, patients, to mothers and fathers and to all others who use our products and services.
- In meeting their needs, everything we do must be of high quality.
- We must constantly strive to reduce our costs in order to maintain reasonable prices.
- Customers' orders must be serviced promptly and accurately. Our suppliers and distributors must have an opportunity to make a fair profit...

As a division of J&J, Janssen's direction is clear: focus on the customer! The pharmaceutical industry must recognize that the customer has changed and continues to change. In the past, the doctors and the fee-for-service approach were key customers. Today, it is the Managed Care Organizations, the Federal Government and Pharmaceutical Business Management Organizations. No matter how specific customers are segmented, they expect pharmaceutical products to be:

- safe and efficacious;
- compliant with regulations;
- available when requested (no backorder);
- reasonably and competitively priced;
- statistically monitored, producing outcomes data (for the pharmaceutical business).

This is not new for the pharmaceutical business. However, another suggestion would be to view the regulatory agencies that review, approve and monitor processes and products. These agencies are also key customers as they can deny approval, significantly affect business with their requirements, and impact product availability (recall, seizure, etc.). Regulatory requirements are as follows:

- management responsibility;
- quality systems established;
- training and competency of personnel;
- documentation;
- data management.

At Janssen, these customer requirements are covered by three categories: quality, cost and time. These need to be discussed as they affect the laboratories—a critical part of the business. Customers expect the product to be safe and efficacious, this is not negotiable! Customer expectations are addressed as follows.

Product life cycle:

- development;
- launch;
- market surveillance.

During product development, methods are developed to ensure that the drug is present in the quantity expected and that it is free from other substances, impurities, etc. When the product is launched, each batch is tested by the

Quality Control Laboratories to ensure that it meets all specifications. The laboratory results in conjunction with process evaluation determine the product disposition. Areas of focus are as follows.

Product disposition:

- Does it meet the required specification? Disso? Content uniformity? Etc.?
- Is it within the normal range? Is it out of trend?
- Does our documentation support our results?
- Is the product properly protected by its packaging to ensure its stability throughout its shelf-life?

Quality programs are necessary that support all of these decisions. The laboratory plays a primary role in determining the product's disposition. If the product is tested incorrectly, it could be a major problem for the patient and/or company. There is no doubt that the role of the laboratory in product disposition is a critical one.

Market surveillance:

- stability testing;
- complaint handling;
- adverse events.

Another role the laboratory plays is monitoring the product during its shelf-life. This is achieved through stability testing of representative batches throughout their expiration period. While this may seem simple, it is not. One must consider global stability requirements, where the product will be sold, and how the product will be shipped and stored. It is one thing to ship the product within the US, it is another to ship it to Zambia or New Zealand or other parts of the world where the climate and storage capabilities may be different. The product dispositioned at release must meet all requirements throughout its shelf-life.

Besides performing stability tests and monitoring all the processes that accompany that, the labs also evaluate complaints or adverse event samples that are returned from the field. While this is not done in all cases, retained samples are tested and may be the determining factor for product withdrawal or recall. Again, there should be no doubt about the role the laboratory plays with respect to the life cycle of the product, assuring its safety and compliance to specification.

The laboratory is not only concerned with testing; there are quality systems that must be built around the testing programs. The need for these cannot be overemphasized. Janssen's quality strategy is depicted in the quality pyramid shown in figure 1.

The first step is to establish quality systems as the foundation, systems, e.g. complaint handling, validation (product, process software, etc.). Janssen is now entering the second phase of automation of systems and data so that information is readily available and actionable. The ultimate goal is to reach the pinnacle of the pyramid; process reliability reduced testing; truly PREVENTIVE **NOT REACTIVE**. To monitor the progress in this journey, quality measures were established.

Quality/key measures:

- number of observations/number of inspections;

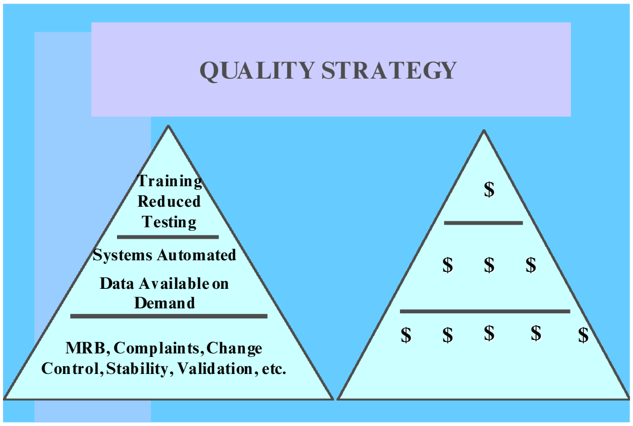

*Figure 1.*

- number of significant observations;
- number of warning letters;
- per cent first pass approval for pre-approval inspections.

The last point listed above is the future of Janssen Pharmaceutica, new products. Every pre-approval inspection that Janssen has had has focused on the laboratory, the lab data and process/product/software validation. Examples of expectations and efforts to excel in this aspect of meeting the FDA, our customer's expectations, are listed as follows.

Efforts to improve first pass approval for pre-approval inspections:

- analytical method technical transfer file (AMTTF);
- training of analysts;
- supervisor ratios;
- documentation;
- instrumentation;
- investigations/deviations;
- audits.

From evaluating the issues of past inspections, these were the main issues that needed improvement. Firstly we deal with method development (figure 2).

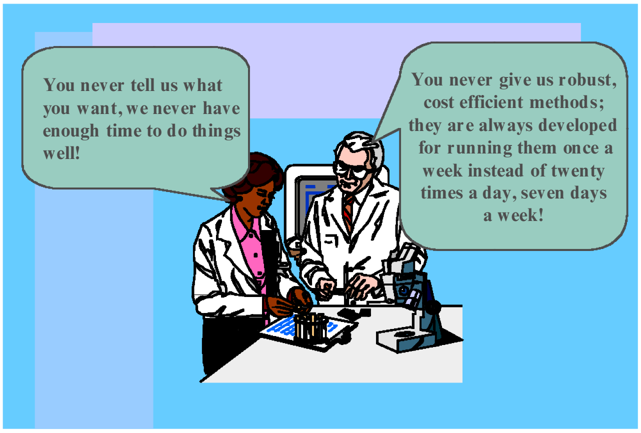

*Figure 2.*

While not everything has been corrected, clear requirements have been developed with checkpoints of evaluation by the development laboratory's key customer, QA/OPS labs. Together the groups came up with the analytical method technical transfer file (AMTTF). This living document is an assimilation of three documents: a method development report; a method validation report; and a method transfer report.

The method development report documents all the pertinent information on how the method was developed, what columns were tested, pH and pressure parameters, etc. The purpose of this document is to serve those who follow as a historical perspective on any issues relevant to this specific compound and method. Later in the product life cycle, if questions arise, the OPS and QA groups can go back into this document and look for the pertinent information.

The method validation report documents all the data that support the key parameters of the method. It contains data confirming that the method is robust and meets the requirements of global compendia.

The method transfer report is the formal documentation that the method has been transferred to the OPS/QA laboratories. This report contains the overall results and executive summary, the transfer protocol and the transfer data supporting that the method can be successfully run at an alternative laboratory location.

Each of these documents has an SOP supporting the requirements and who must approve them. This effort established the foundation for improving communication within internal groups, clarified the expectations of the internal customers, and clearly meets the expectations of external customers, the FDA. As the product line matures, OPS/QA then work to refine the methods as appropriate to improve efficiencies and reduce cost.

Laboratory training: Laboratory Analytical Training and Certification Course:

- co-developed by J&J and Drew University;
- two weeks of intense training (classroom and hands-on);
- test results required for certification;
- remediation training;
- future programs.

Simultaneously to developing the AMTTF process, under the guidance of Hank Avallone, Executive Director of Compliance, and Nancy Corkum, COO of IRI, J&J worked with key lecturers and Drew University to develop an analytical chemistry Analyst Certification Program. The Program brings in lecturers to present compliance and technical information that the analyst needs to know to work within a compliant pharmaceutical laboratory. The Program spans 2 weeks, and includes verbal and written tests, and application and workbook evaluation. The goal is for all analysts to receive a certification on a periodic basis. For those few analysts who do not certify, a remediation program has been developed. Future enhancements to this program include a program specializing in HPLC technology and managerial training.

Additionally, there is a need for a strong supervisor/analyst relationship. Janssen's current ratio of analysts to supervisors is 8:1. While this initially seemed costly to the organization, it has begun to show payback in attention to addressing instrumentation and general laboratory issues providing immediate feedback to those new analysts who are learning the methods, processes and expectations. This ratio provides for review of data, review of training needs, reasonable time for personnel development and, in general, good laboratory management practices. By implementing this approach, it was discovered that some of the instrumentation in the lab was not appropriate for the type of work being performed. It was also discovered that training approaches were not consistent with laboratory practices. These are the types of issues that go unresolved or unnoticed until they become a problem if the analyst:supervisor ratio exceeds 10:1.

Three key areas of laboratory documentation received attention in the corrective action review:

- training records;
- data review and archival;
- data management.

Analysts had been trained along the lines of laboratory technology: dissolution, assay, etc. Upon closer scrutiny, it was discovered that this was not sufficient, it was necessary to ensure that analysts were trained on each specific method and SOP. This provided assurance that all aspects of testing were understood; there could no longer be reasons, e.g. 'I didn't know' or 'I wasn't aware'. Areas, e.g. safety, methodology and data calculations are all included in this training.

The data are evaluated by the analyst and then by a peer reviewer. After passing this process, the data are reviewed again by the supervisor. Prior to entering the data into the various reports (stability, process characterization or validation), it is verified again against the original data to ensure that typos and transposition errors are minimized. All data are then filed for easy retrieval.

Instrumentation also presented itself as an issue. Much of the instrumentation was outdated and unable to perform as necessary for some of the more complex methods. This resulted in a sizeable investment, in excess of $1MM. In order to maintain a competitive advantage in this area, new instrumentation is constantly being evaluated for improvements.

Last, but certainly not least, it was necessary to focus on the quality and timeliness of investigations. Although analysts were trained and up-to-date instrumentation was provided, out-of-specification and out-of-trend results were still encountered from time to time. Janssen's record on addressing investigations adequately and in a timely manner was poor. It was necessary to refocus and apply areas of scrutiny by the FDA investigators and, from a business perspective, inventory levels suffered. General training programs have been held on how to write an investigation, the key elements of an investigation, and the expected timeliness of closure. Although all the goals have not yet been met, progress is being made.

To ensure goals are met and key areas of concern are addressed, a periodic laboratory audit program was established, whereby the Compliance Group performs audits no less than once every 6 months. These audits are generally unannounced or announced within 2 or 3 days of the audit. This process not only ensures that attention stays focused, but also prepares the laboratory personnel for audits from the customer, the FDA. How to answer questions and how to present data are covered, the following words are avoided as far as possible:

- contaminated;
- failure;
- violation;
- backorder/cost savings;
- business decision;
- possibly/may/might.

The FDA expects that products, methods and processes are understood, and that decisions are made based upon written procedures and science, not on business needs. They also expect definitive answers to out-of-specification or out-of-trend investigations, are not satisfactory!

Additionally, the Compliance Group performs a pre-PAI readiness audit to ensure the labs are prepared in the areas specifically associated with the new product awaiting approval.

From experience at Janssen, these are areas that are focused on. To remain alert to the FDA, the customer's thinking, FDA citations of other non-J&J companies were investigated. For example:

United States versus Biocraft Laboratories (7/94):

- stability plan;
- failure investigation procedure;
- analyst training;
- adequate methods, facilities and controls;
- adequate methods, equipment, record keeping and controls at each laboratory.

The FDA guide to inspections of Pharmaceutical Quality Control Laboratories, issued in July 1993 was reviewed. This document stressed keeping SOPs current. Current areas of focus are raw data management, data reprocessing and integration, and in-depth review of the CFR. It is hoped that improvement will continue in meeting the requirements of internal and external customers, yielding high-quality product, consistently. In addressing the compliance aspects, it is expected that confidence will be built with the customer, the FDA, such that when they inspect, their findings are not significant. If a good compliance profile is maintained, the ultimate goal will be approval of new products without an inspection. The business impact here is quite significant in time and dollars (speed to market).

As reported in the Wall Street Journal and the Pink Sheets, last month, Steris, a division of Shein Pharmaceuticals had a very difficult PAI inspection. Government agents halted production. The stock plunged 39% from $32 to 14 $\frac{3}{8}$. It affected the scheduled launching of 11 of 23 new products. The warrant for the seizure noted uncorrected problems, e.g.:

- failure to maintain sanitized equipment;
- failure to establish adequate procedures for production and laboratory controls;
- failure thoroughly to investigate unexplained discrepancies in batches of drugs.

These reviews of current regulatory actions helped to reinforce commitment to understanding and meeting the customer's requirements. Janssen Pharmaceutica cannot afford this type of business impact.

The next category of measurement is cost. Key measurements in this category are as follows:

- cost of quality organization as a percentage of sales;
- productivity, tests per analyst;
- headcount;
- cost of quality as a percentage of cost of goods sold;
- cost of lot disposition.

Because 45% of the headcount is laboratory related, the cost of the quality organization is for the most part a laboratory issue. As previously mentioned, new equipment was purchased at a cost of over $1MM. The depreciation on this investment will remain for several years. System improvements, e.g. LIMS, data management, Y2K assurances, etc. are all cost drivers for the laboratory and quality organization. It is clear from a management perspective that Janssen must invest; however, these investments must be well planned and balanced with the business growth. As previously mentioned, Janssen is somewhat unique with headquarters in Belgium, production site in Puerto Rico and administrative offices in New Jersey. Methods are developed in Belgium, evaluated and transferred to New Jersey where stability and support process start-up are performed, and then transferred to Puerto Rico for production start-up. In the past, methods were developed with a European perspective and then modified for those products being sold in the US. As a competitive global company, this approach can no longer be afforded; methods must now be developed with a global mindset.

Once production starts up and forecasts are validated, the Titusville laboratories move into a method improvement mode, looking for efficiencies and cost savings. Recently, a robotics program for high-volume products was embarked upon. The first experience took over nine months and was almost given up. The Titusville group working with the Puerto Rico Labs and Zymark developed the robotic methods for dissolution, assay and content uniformity of one of the high-volume products. The SOPS were developed and approved, methods were transferred, dossiers were prepared and approved, and training completed. At last, implementation was imminent. The robot was fondly named 'R2D2'. Within 2 weeks of implementation, low assay was experienced in one of the samples. The sample was redone and the root cause of the problem could not be identified. The laboratory analysts and supervisor of this, the first robotic approach, were stunned. Would the robot be unreliable? Would all the work put into this programme be for nothing? The analysts, Raul Homs and Lillian Vazquez, would not give up. They scratched their heads, called Zymark, the supplier, and still no root cause was determined. These analysts were persistent. They borrowed the company video camera and set it up to run for 24 h.

They were so dedicated, they came in at the off hours to change the tapes.

The next morning, the tapes revealed the problem. The tablet was vacuumed up and plugged the canula. This was the root cause, and it was caught and documented on tape! The problem could then be explained, the batch dispositioned, and, by raising the canula, a recurrence prevented. The robotics programme was again back on track. The implementation of four additional product-focused robotics programmes within the next nine months is planned.

Robotics savings on cost:

- four analysts;
- reduced cycle time by 48 h;
- improved compliance.

The first robotics experience saved the company well in excess of \$300 000 annually. Additionally, the headcount saved meant that staff were able to be reassigned to bring in outside testing at a cost avoidance of \$500 000 annually. Next could be the installation of an LIMS system, looking at NEAR IR programs and others that will facilitate laboratory efficiencies so that productivity can be increased and cost decreased.

Lastly, the key customer measurement is time. It is necessary to be vigilant in challenging laboratory cycle time. Here is where programmes established can affect headcount, inventories and compliance—all impacting cost and time-to-market. Key measures for time are:

- cycle time for raw material testing: sample-in to sample disposition;
- cycle time for bulk product testing: sample-in to sample disposition;
- cycle time for finished goods testing: sample-in to sample disposition:
- cycle time for documentation review and product disposition.

Lower cycle time means less inventory, less inventory means less carrying costs and improved customer satisfaction; the product is available when the customer wants it—no backorder. By focusing on the metrics and reporting out on a quarterly basis, issues are brought forward and successes are rewarded.

As previously mentioned, Janssen compete for best practice and benchmark position internally and externally. Internally, Janssen 'peel the onion' on the data to understand who is doing best and why and then implement these best practices across all of the sites. Similar data are compared with competitors via a third party. This helps to understand how Janssen are doing in relation to the rest of the industry, and prompts competitiveness and innovation to improve.

The Credo advised that the second order of priority is the employee, the greatest resource and competitive weapon.

Janssen's Credo:

- We are responsible to our employees, the men and women who work with us throughout the world. Everyone must be considered as an individual.
- We must respect their dignity and recognize their merit.
- They must have a sense of security in their jobs.
- Compensation must be fair and adequate, and working conditions clean, orderly and safe.
- We must be mindful of ways to help our employees fulfil their family responsibilities.
- Employees must feel free to make suggestions and complaints.
- There must be equal opportunity for employment, development and advancement for those qualified.
- We must provide competent management; their actions must be just and ethical.

Janssen's goal is to attract, develop, promote and retain the best employees. From a laboratory perspective and in this current job market, this is not an easy task. Several approaches are being experimented with in this area.

Human resource excellence:

- college recruiting programme;
- technical and managerial ladder;
- reward systems;
- rotational opportunities;
- training;
- facilities/instrumentation

While salary is an important part of hiring and retaining employees, there are other areas of importance. People want to be developed, learn what is the latest in instrumentation, become a business partner and understand how their contributions impact the business. At Janssen, the need to hire experienced lab personnel who can 'hit the ground running' is balanced with entry-level positions. A college recruitment programme is being embarked upon for all levels of college graduate. Alliances with Drew University, Seton Hall and Temple are being cultivated. Janssen are working with Seton Hall to bring a Master's Degree programme to one of the J&J facilities in NJ so that personnel can continue their education with minimal travel and expense. A similar programme is also being pursued with Temple focused on QA/RA Master's Degree curricula. A dual ladder programme is being prepared. In the laboratory, if one chose to stay at the bench and continue to develop and contribute, there was no place to go. It was finally realized that not all scientists are managers! This needs to be recognized, as well as the fact that a key chemist or scientist contributes equally as much as a senior manger. As a result, a technical ladder is being developed that would provide financial and title recognition for those who do not wish to enter the managerial ranks, but contribute on a scientific basis.

Reward systems are in place. Often in the past, new product teams would be rewarded when a new product was approved. Recognition for the contribution of the labs was omitted. They are now integrated into the team structure and are recognized along with the rest of the team for their commitment and contributions. In addition, when major method breakthroughs occur, problem solving or method improvements are brought forward, an additional recognition is offered which ranges from 1 to 5% of salary.

For those high-potential people, the opportunity for job rotation to other J&J companies within the US, in Puerto Rico or abroad is offered. This provides growth scientifically and culturally making the employee more valuable to the Corporation as his or her span of influence is usually expanded during and after this experience.

Human Resource excellence cannot be achieved without training. All employees want to improve themselves and their ability to contribute. At Janssen, Hank Avallone, the Executive Director of Compliance, worked with several sister company contributors to develop the GMP 13 Compliance Manual. This manual focuses on the compliance requirements for the Janssen Companies. Laboratory controls, laboratory management, stability, deviations, etc. make up a significant part of the manual. Hank also arranges an annual meeting with the FDA both in the US and in Puerto Rico. This gives the analysts the opportunity to interface with the FDA, hear the current thinking of customers, and ask questions that would not be possible during a specific inspection.

Facilities and instrumentation are also key to attracting and retaining a strong employee workforce. A pleasant, safe work area, with basic needs, child care, on-site exercise facility, cleaners, bank, etc. are all part of the Janssen effort to hire and retain the best employees.

Janssen's Credo:

- We are responsible to the communities in which we live and work, and to the world community as well.
- We must be good citizens—support good works and charities, and bear our fair share of taxes.
- We must encourage civic improvements and better health and education.
- We must maintain in good order the property we are privileged to use, protecting the environment and natural resources.

After customer and employee needs are fulfilled, the Credo requires focus on the community. We are encouraged to respect business neighbours and support the needs of the communities in which we live and work. Again, from a laboratory perspective, it is necessary that all OSHA, EPA and DEA, etc. requirements are met. Laboratory solvents, waste, etc. must be stored properly and discarded according to local and federal laws. As with compliance, internal Janssen audits as well as corporate audits are performed in these areas to ensure compliance with the regulations. Overall safety, audit, OSHA and EPA inspection results are reviewed with the Management Board at least annually.

Employees are also encouraged to support community agencies, e.g. United Way, Walk for the Cure, Blood Drives, Road Cleanup, Habitat, etc.

Janssen's Credo:

- Our final responsibility is to our stockholders.
- Business must make a sound profit.
- We must experiment with new ideas.
- Research must be carried on, innovative programs developed and mistakes paid for.
- New equipment must be purchased, new facilities provided and new products launched.
- Reserves must be created to provide for adverse times.
- When we operate according to these principles, the stockholders should realize a fair return.

Lastly, if all the above priorities are delivered—customer, employees and community—the return on investment for the stockholders will be favourable and more investment for new products, facilities and overall business growth will be encouraged.

### Conclusion

Finally, management needs to be mentioned. Too often the laboratory is neglected; its personnel and contributions to the business and bottom line. Attention is not usually paid until there is a negative event. To become and stay leaders in industry, it is essential to pay attention.

Management responsibility:

- authorization of resources;
- establishment of timeframes;
- correction of objectionable conditions.

Management are responsible for authorizing resources, establishing timeframes and addressing objectionable conditions. These issues cannot be delegated or ignored. The laboratory plays a key role in:

- product development;
- time to market;
- product quality assurance;
- product cost;
- customer satisfaction.

Everyone has the responsibility to pay attention and challenge how things can be done faster, better, cheaper, safer. The laboratory is no exception and can only succeed with the support of the management.

### Acknowledgements

I would like to thank John Ortiz, Janssen's previous QA/QC Director of the Puerto Rico facility, for having the vision to invest in the robotics programme, and the supporting people at both Puerto Rico and Titusville: Maria Pallares, Raul Homs, Lillian Vazquez, Tim Diehl and Darryl Roberts who are making it happen.

